# Three Layers of Intestinal γδ T Cells Talk Different Languages With the Microbiota

**DOI:** 10.3389/fimmu.2022.849954

**Published:** 2022-03-24

**Authors:** Francesca Rampoldi, Immo Prinz

**Affiliations:** ^1^ Institute of Medical Microbiology and Hygiene and Research Center for Immunotherapy (FZI), University Medical Center, University of Mainz, Mainz, Germany; ^2^ Institute of Immunology, Hannover Medical School, Hannover, Germany; ^3^ Institute of Systems Immunology, Hamburg Center for Translational Immunology (HCTI), University Medical Center Hamburg-Eppendorf, Hamburg, Germany

**Keywords:** gut epithelia, lamina propria (LP), γδ T cells, IEL intra-epithelial lymphocyte, Peyer’s patch

## Abstract

The mucosal surfaces of our body are the main contact site where the immune system encounters non-self molecules from food-derived antigens, pathogens, and symbiotic bacteria. γδ T cells are one of the most abundant populations in the gut. Firstly, they include intestinal intraepithelial lymphocytes, which screen and maintain the intestinal barrier integrity in close contact with the epithelium. A second layer of intestinal γδ T cells is found among lamina propria lymphocytes (LPL)s. These γδ LPLs are able to produce IL-17 and likely have functional overlap with local Th17 cells and innate lymphoid cells. In addition, a third population of γδ T cells resides within the Peyer´s patches, where it is probably involved in antigen presentation and supports the mucosal humoral immunity. Current obstacles in understanding γδ T cells in the gut include the lack of information on cognate ligands of the γδ TCR and an incomplete understanding of their physiological role. In this review, we summarize and discuss what is known about different subpopulations of γδ T cells in the murine and human gut and we discuss their interactions with the gut microbiota in the context of homeostasis and pathogenic infections.

## Introduction

**Figure 1:**
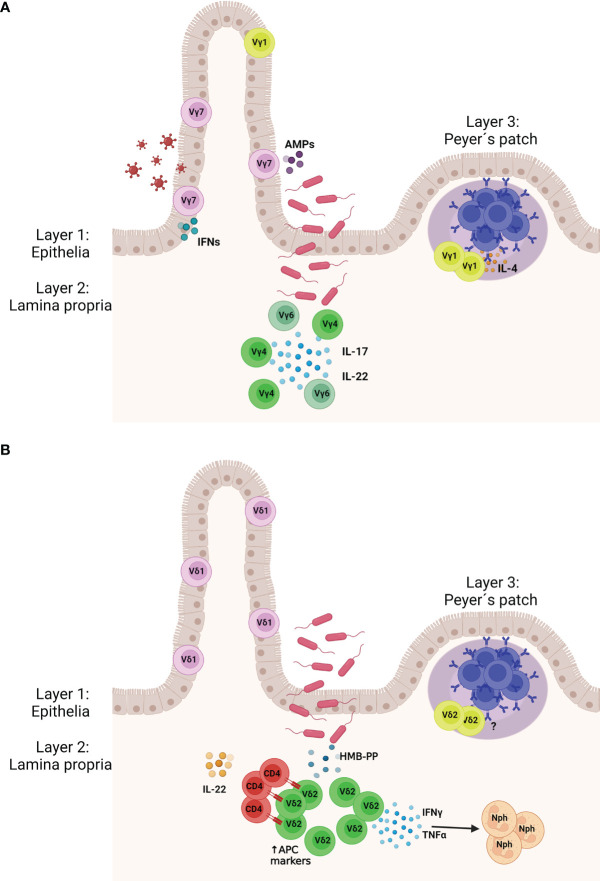
Murine **(A)** and human **(B)** γδ T cell protect the gut tissue against pathogens. (Layer 1) γδ IELs patrol the gut epithelia and are interspersed between the epithelial cells. (Layer 2) Upon pathogen invasion lamina propria (LP) γδ T cells expand and produce cytokines to activate the locally the immune system. In mice LP γδ T cells express the Vγ4 and Vγ6 chains and mainly produce IL-22 and IL-17. In humans, LP γδ T cells express the Vδ2 chain and upon stimulation with (E)-4-hydroxy-3-methyl-but- 2-enyl pyrophosphate (HMB-PP) produced by bacteria, they release IFN-γ and TNF-α, which attract neutrophils. Vδ2^+^ γδ T cells may also express markers of antigen presenting cells and present antigen to CD4 T cells, which start producing IL-22. (Layer 3) Specific populations of γδ T cells are found in the Peyer´s patches where they support the humoral immunity, including the production of the immunoglobulin **(A)** AMPs, antimicrobial peptides; Nph, neutrophil. Created with BioRender.com.

The mucosal immune system represents the first barrier of the body against pathogenic invaders. At the same time, it is responsible for maintaining the symbiotic relationship between the microbiota and the host. Although the microbiota have beneficial functions for the host, they also represent a threat to penetrate the mucosal barrier. Thus, a tight regulation of tissue integrity and a rapid immune response are required (1). The intestinal epithelium consists of only a single layer of epithelial cells that separates the intestinal lumen from the lamina propria (LP), the mucosal tissue situated underneath (**Figure 1**). The epithelium forms either crypts or villi where water and nutrients are absorbed. To fulfill its multiple functions, the mucosal immune system is composed by extremely heterogeneous populations of leucocytes. Indeed, next to γδ T cells and αβ T cells, the intestinal epithelium and the LP harbor plenty of immune cells including B cells, innate lymphoid cells (ILC)s, macrophages, and dendritic cells (2).

γδ T cells express on the surface a γ and a δ chain, which together form the γδ TCR. They are enriched in the peripheral tissue, but represent only a small percentage of all T cells in the blood (1-5% of total T cells in humans) (3, 4). In mice, γδ T cell subsets are mainly defined by the expression of their γ chain (Heilig and Tonegawa nomenclature) (5), e.g. Vγ1^+^, Vγ4^+^, or Vγ7^+^ γδ T cells; whereas in humans they are grouped according to their δ chain, e.g. Vδ1^+^ or Vδ2^+^ γδ T cells. γδ T cells are highly abundant in the gut, where they display diverse phenotypes and functions (6). Among them, γδ intestinal intraepithelial lymphocytes (IEL)s are localized between the epithelial cells (7, 8) (**Figure 1**). They are mainly tissue resident (9) and their phenotype is shaped by the local environment (10, 11). Beside γδ IELs, other γδ T cells subsets populate the LP and are termed γδ LPLs. They can re-circulate in the blood or be tissue-resident (10). Finally, Peyer´s patches (PPs) are also home to γδ T cells, in particular to a specific subpopulation important for the humoral response and the production of the immunoglobulin A (IgA) (12, 13) (**Figure 1**).

In this review, we focus on the different subsets of intestinal γδ T cells, including tissue-resident and circulating populations, which populate the murine and human gut and PPs. We also discuss their interactions with the gut microbiota in the context of homeostasis and pathogenic infections.

## First Layer: γδ Intraepithelial Lymphocytes

In this paragraph, we first revisit the functions, origin, and tissue-development of γδ IELs and then discuss their relation to the microbiota at steady state and during infections.

IELs are fundamental for preserving the tissue integrity of the gut, maintaining the symbiosis with the microbiota, and providing continuous surveillance of the intestinal epithelium (14–16).

γδ IELs belong to the natural or type B IELs and express the homodimer CD8αα co-receptor (17). 20% - 30% of human IELs express the γδ TCR; while in mice their frequency makes up to 50% - 60% of all IELs (11, 18). Murine γδ IELs populate the intestine during the perinatal period and mainly express the Vγ7 chain. Although they may appear like an invariant population, their TCR repertoire is still very diverse, endowing them with the potential to recognize a wide array of antigens (6, 19).

It has been suggested that γδ IELs may partially develop extrathymically, as a few γδ IELs still develop in nude mice, which lack a thymus (18, 20). However, it is likely that IEL precursors develop in the thymus before entering the gut (21, 22). Interestingly, intestinal epithelial cells are able to produce IL-7, a cytokine important for intrathymic T cell development (23, 24).

Their recruitment to the intestinal compartment is mediated by the expression of CCR9, which recognize CCL25 produced from intestinal epithelial cells (25, 26) and their accumulation into the epithelium is regulated *via* binding of integrin αEβ7 (CD103) on IELs with E-cadherin expressed on enterocytes (27). In the epithelia, dietary compounds, such as aryl hydrocarbon receptor (AhR) ligands, and IL-15 produced by epithelial cells, are essential for the maintenance of human and murine γδ IELs (28–33). Importantly, they are locally shaped by specific molecules, the butyrophilin-like (Btnl) subfamily of B7 genes (11, 34). In the gut of mice, *Btnl1*, expressed by the epithelial cells of the villi, selectively promote the maturation and expansion of Vγ7^+^ γδ T cells (11) and, together with Btnl6 induce a TCR-dependent stimulation of these cells. Interestingly, different Btnl heterodimers had diverse effects on IELs with different TCRs, indicating that they may fine-tune the IEL numbers, composition, and function in the gut (34).

In humans, the Vδ2^−^ γδ T cell subset is enriched in the intestinal tissue and is also rather heterogeneous (35) (**Figure 1B**). Similarly to mice, the human gut epithelia express the closely related proteins BTNL3 and BTNL8 which promote the local colonic expansion of a specific Vγ4^+^ γδ T cell subset (11). Interestingly, during chronic inflammation driven by celiac disease, loss of BTNL8 expression by the gut epithelium was accompanied by the reduction of Vγ4Vδ1^+^ γδ IELs and by the generation of IFN-γ producing Vδ1^+^ γδ IELs whose TCR lacked reactivity against BTNL3 and BTNL8 (36). Therefore, also in human the BTNL molecules have a critical role in shaping tissue-resident γδ IELs.

### Crosstalk of γδ IELs With the Microbiota

Unexpectedly, the gut microbiota does not influence the number of intestinal γδ IELs, as germ-free (GF) and specific pathogen-free (SPF) mice show comparable numbers of these IELs (18). However, their functions and motility behavior can be conditioned by the microbiota (1). γδ IELs are highly mobile and there is one IEL every ca 5 - 10 epithelial cells (17). IELs patrol the basement membrane by migrating between adjacent epithelial cells, a behavior called “flossing” that has been captured by intravital microscopy using transgenic mice with green fluorescent γδ T cells (9, 11, 17–20). More recently, it has been shown that γδ IELs exhibit a microbiota-dependent localization and movement pattern. Infections of pathogenic bacteria or protozoa induced an active response by γδ IELs, which resulted in increased intraepithelial cell scanning, expression of antimicrobial genes and metabolic switch towards glycolysis (33, 37). These changes were dependent on the pathogen sensing by intraepithelial cells through the MyD88 signaling (33)

This pathway seems to be involved also in the ability of γδ IELs to express several innate antibacterial effectors, including regenerating islet-derived protein 3γ (REG3γ) or chemotactic cytokines in response to a resident bacterial pathobiont, which is able to penetrate into the cells of the host (38) (**Figure 1A**). In fact, this bacterial stimulation is mediated by intestinal epithelial cells *via* the activation of MyD88 signaling, indicating that γδ IELs receive microbe-dependent cues directly from epithelial cells (38). Accordingly, the absence of γδ T cells was associated with an increased bacterial burden in *Tcrd* ^–/–^ mice following acute dextran sulfate sodium (DSS)-induced intestinal damage or invasion by other pathogens (39–41). All together, these data reveal a dialogue between the microbiota and γδ IELs, which specifically respond to invading bacteria, both resident (pathobionts) or exogenous (38). Besides bacteria, γδ IELs were shown to protect the intestinal epithelial cells from murine norovirus infections by promoting the antiviral response, dependent on production of type I, II and III IFNs by IELs (32) (**Figure 1A**). Additionally, activated intestinal IELs increased the resistance of intestinal epithelial cells to viral infection (32). In that study, mice were first treated with anti-CD3 or control antibodies and then orally infected with a norovirus. The level of infection was reduced in mice pre-treated with anti-CD3 antibodies, suggesting that the pre-activation of IELs *via* TCR engagement enhance the resistance to norovirus infections (32).

Another characteristic of the γδ IELS is that they exist in a so-called “activated yet resting” status (17) describing a chronically activated phenotype (42). They homogeneously express the activation marker CD69, NK cell associated-molecules like 2B4/CD244, NKG2A, NKG2D, NKp46 and NK1.1 (28, 43), and some cytolytic genes such as granzymes A and B, perforin, and Fas ligand, indicating a cytotoxic activity towards pathogens and infected cells as well as potential to trigger apoptosis (29, 30). However, evidence for direct cell lysis by γδ IELs *in vivo* is still elusive (1, 31).

In summary, there is a coordinated crosstalk between the microbiota, epithelial cells and γδ IELs, which support the maintenance of homeostasis with the intestinal microbiota and the epithelial barrier defense.

## Second Layer: Lamina Propria γδ Lymphocytes

LP is a thin layer of connective tissue, which is situated beneath the epithelial cells and contains different γδ T cell populations that are influenced by the microbiota in distinct ways. In contrast to the γδ IELs, which never produce IL-17A, LP-resident γδ T cells can readily produce IL-17 and other “type-3” cytokines (42). Of note, the frequencies of γδ17 T cells in the LP are decreased in GF mice or in mice treated with antibiotics, implying that specific microbiota promote their differentiation or expansion *in situ* (32). Specifically, signaling through the guanine nucleotide exchange factor VAV1, required in the TCR signal transduction, is essential for the expansion of this pool of LP γδ17 T cells, indicating an involvement of the TCR in the interaction between intestinal microbiota and LP γδ17 T cells (32). In this scenario, macrophages and dendritic cells may produce IL-1 and IL-23, which then induces the production of IL-17 by LP γδ17 T cells (32, 33). These γδ17 T cells share comparable features with Th17 cells, such as the expression of chemokine receptor 6, retinoid orphan receptor (RORγt), AhR, and IL-23 receptor (37, 44, 45). So far, a specific bacterial species that is able to expand LP γδ17 T cells has not been recognized (46). However, their dependency on microbiota could also be indirect *via* production of IL-10 by Treg cells (47) or regulated by the production of short-chain fatty acids by the microbiota themselves (48).

In mice, a distinct subpopulation of LP γδ T cells accumulate in the intestinal epithelium and associated mesenteric lymph nodes after exposure to *Listeria monocytogenes* (*Lm*) (49). These LP γδ T cells appear to be very different from the ones previously described as they form a stable long-lived memory population. They express the Vγ6Vδ1 chains and are able to produce IFN-γ and IL-17 at the same time. Interestingly, they quickly expand after second exposure to oral *Lm* but not oral *Salmonella* or intravenous *Lm*, indicating a specific dependence on *Lm*, although their TCR ligand is not clear yet (50–52). These findings point to an adaptive-like tissue-specific accumulation of innate γδ17 T cells after bacterial infections.

In addition, in the mouse, the abundance of LP γδ T cells varies along the gastrointestinal tract. In the colon a specific population of γδ17 T cells has been reported to express the Vγ4 chain as well as CCR6 and to be restricted to the innate lymphoid follicles (53).

Finally, besides IL-17, other subsets of LP γδ T cells and innate lymphoid cells type 3 (ILC3) can produce IL-22 (33, 54–56), which controls the release of antimicrobial peptides and enforces tight junctions between enterocytes to limit bacterial dissemination and intestinal inflammation (57, 58).

In humans, 1-5% of the total T cells in the gut are Vγ9/Vδ2 cells (59). Conversely to γδ IELs, LP γδ T cells are recruited from the peripheral blood and proliferate locally in order to preserve the local pool mainly constituted by Vδ2^+^ cells (7) (**Figure 1A**). They recognize microbiota- associated metabolites; specifically, they are triggered by phosphoantigens like HMB-PP expressed by bacteria (60–62). Microbe-responsive Vγ9/Vδ2 cells acquire a gut-homing phenotype by increasing the level of the marker CD103, express antigen presenting cells markers and influence the expression of IFN-γ by autologous colonic CD4^+^ T cells (59) (**Figure 1B**). In line with this data, Vδ2^+^ T cell were recruited to the gut and expanded after injection of HMB-PP into macaques (7, 63) (**Figure 1B**). Moreover, in order to support the mucosal defense, Vγ9/Vδ2 cells activated by bacterial phosphoantigens may recruit neutrophils to the site of invasion, stimulate CD4^+^ T cells to release IL-22, and promote the production of the IL-22-inducible antimicrobial protein calprotectin by the epithelial cells without affecting the production of IL-17 (64, 65). However, γδ17 T cells are quite abundant in infants and may be involved in the protection of the mucosal barrier during neonatal life (66, 67).

It is worth to mention that γδ17 T cells in other organs can respond to intestinal microbial cues (10, 47, 68, 69) and they have been extensively discussed elsewhere (46).

In summary, conversely to the γδ IELs, LP γδ T cells comprise a big variety of different subpopulations which are very distinct between humans and mice; however, they have a common goal, to support and promote the mucosal immune system in response to invading pathogens.

## Third Layer: γδ T Cells In Peyer´S Patches

PPs constitute one of the major components of the mucosal-associated lymphoid tissue and are located along the small intestine. In adult humans, between 100 – 200 PPs can be found in the small intestine (70), whereas mice have approximately 6 – 12 PPs (71). Peyer’s patch formation is profoundly affected by the production of IL-7 from intestinal epithelial cells (24).

Because of a constant stimulation by the nearby microbiota, germinal centers (GCs) in PPs are continuously formed and maturation of high affinity B cells is achieved through somatic hypermutation and class switch recombination, in particular towards the IgA isotype. In fact, IgA is the most abundant antibody of the gut and is mainly secreted by plasma cells generated in the GCs to maintain the homeostasis with the microbiota (72, 73). Our lab recently demonstrated that γδ T cells (mainly Vγ1^+^ γδ T cells) can be found in PPs and that they localize inside and at the border of the GCs (12). In particular, we showed that a restricted subset of Vγ1^+^ T cells is able to produce IL-4, thereby inducing B cell isotype switch towards IgA (**Figure 1A**). Their absence altered the development of IgA^+^ GC B cells not only at steady state but also in the context of *Salmonella* infection (12). The influence of Vγ1^+^ T cells on IgA was also shown at steady state in Vγ1*^−/−^* mice, where the concentration of IgA^+^ B cells was diminished compared to WT mice (74). Also, *Tcrd^−/−^* mice presented an even stronger reduction of IgA levels in serum, saliva, and fecal samples after exposure to tetanus and cholera toxin (75). Interestingly, IgM and IgG concentrations were not affected, further corroborating a specific role for PP γδ T cells in the production of IgA (75). These data leave a lot of open questions, in particular regarding the repertoire and the specificities of the γδ TCR. Do Vγ1^+^ T cells recognize specific signals/antigens *via* their TCR or does their help rely on other signaling molecules (12)?

Human PPs also harbor a small percentage of γδ T cells, mainly of the Vδ2^+^ subset, and thus different from the γδ IELs (76) (**Figure 1B**). Interestingly, a fraction of the PP γδ T cells is CD62L^+^, probably recruited from the blood while another fraction is CD45R0^+^ and possibly antigen-primed (76). Weather these γδ T cell subpopulations contribute to the humoral response also in humans is still an open question.

## Concluding Remarks

γδ IELs, LP γδ T cells and PP γδ T cells are distinctly shaped by the intestinal microenvironment and by the microbiota. However, how they in turn shape the microbiota and the microenvironment is not completely understood. Further work is necessary to understand the intricate and intriguing interplay between the microbiota, epithelial cells and immune system at local level as well as the effect in other organs. In order to achieve that, careful experimental design should be carried out in order exclude confounding environmental factors and housing conditions (46).

In sum, intestinal γδ T cells act synergistically with the local immune system and epithelial cells to preserve the symbiosis with the gut microbiota and they contribute to the immune responses against invading pathogens directly at three levels: in the epithelial lining, in the LP, and in PPs. Thus, understanding the crosstalk of γδ T cells with the microbiota and identifying the elusive antigens of their TCR in the gut may provide novel therapeutic targets for the treatment of intestinal pathologies.

## Author Contributions

FR wrote the first draft of the manuscript. IP wrote sections and revised the manuscript. All authors contributed to manuscript revision, read and approved the submitted version.

## Funding

The researchers received funding from Hannover Medical School (HILF I Hochschulinterne Leistungsförderung), number 79228008 (to FR), and from the Deutsche Forschungsgemeinschaft, grants PR727/11-2, PR727/13-1 and SFB900/project ID158989968 (to IP).

## Conflict of Interest

The authors declare that the research was conducted in the absence of any commercial or financial relationships that could be construed as a potential conflict of interest.

## Publisher’s Note

All claims expressed in this article are solely those of the authors and do not necessarily represent those of their affiliated organizations, or those of the publisher, the editors and the reviewers. Any product that may be evaluated in this article, or claim that may be made by its manufacturer, is not guaranteed or endorsed by the publisher.
